# Specific N-glycans of Hepatocellular Carcinoma Cell Surface and the Abnormal Increase of Core-α-1, 6-fucosylated Triantennary Glycan via N-acetylglucosaminyltransferases-IVa Regulation

**DOI:** 10.1038/srep16007

**Published:** 2015-11-05

**Authors:** Huan Nie, Xia Liu, Yubao Zhang, Tingting Li, Chao Zhan, Wenjuan Huo, Anshun He, Yuanfei Yao, Yu Jin, Youpeng Qu, Xue-Long Sun, Yu Li

**Affiliations:** 1 School of Life Science and Technology, Harbin Institute of Technology, Heilongjiang, China; 2 Department of Chemistry, Cleveland State University, Cleveland, OH 44115, USA; 3 Tumor Hospital of Harbin Medical University, Harbin, 150081, Heilongjiang, China; 4 School of Basic Medical Sciences, Mudanjiang Medical University, Heilongjiang, China

## Abstract

Glycosylation alterations of cell surface proteins are often observed during the progression of malignancies. The specific cell surface N-glycans were profiled in hepatocellular carcinoma (HCC) with clinical tissues (88 tumor and adjacent normal tissues) and the corresponding serum samples of HCC patients. The level of core-α-1,6-fucosylated triantennary glycan (NA3Fb) increased both on the cell surface and in the serum samples of HCC patients (*p* < 0.01). Additionally, the change of NA3Fb was not influenced by Hepatitis B virus (HBV)and cirrhosis. Furthermore, the mRNA and protein expression of N-acetylglucosaminyltransferase IVa (GnT-IVa), which was related to the synthesis of the NA3Fb, was substantially increased in HCC tissues. Knockdown of GnT-IVa leads to a decreased level of NA3Fb and decreased ability of invasion and migration in HCC cells. NA3Fb can be regarded as a specific cell surface N-glycan of HCC. The high expression of GnT-IVa is the cause of the abnormal increase of NA3Fb on the HCC cell surface, which regulates cell migration. This study demonstrated the specific N-glycans of the cell surface and the mechanisms of altered glycoform related with HCC. These findings lead to better understanding of the function of glycan and glycosyltransferase in the tumorigenesis, progression and metastasis of HCC.

The liver is an active secretory organ and is one of the organs of the secretory glycoproteins. Hepatocellular carcinoma (HCC) is one of the most common cancers and has very high mortality due to its difficult early diagnosis and limited treatment. HCC was responsible for approximately 748,300 new liver cancer cases and 695,900 cancer mortalities worldwide in 2008, ranking as the second most frequent cause of cancer death^1^. Half of these cases and deaths were estimated to occur in China. However, HCC incidence rates are increasing in many parts of the world, including the United States and Central Europe^2^. Therefore, it is urgent to study the molecular mechanism of HCC and to find therapeutic targets and diagnosis mechanisms for effective medical treatment.

Cell surface glycans play an important role in intercellular and intracellular processes, including cell adhesion and development, cell recognition, and cancer development and metastasis^3^. Changes in cell surface glycosylation modulate cellular activity. Aberrant glycosylation of the cellsurface is associated with the malignant transformation of normal cells. Tumor-cell-surface glycans are closely associated with tumor-cell migration, adhesion, and metastasis^4^. The tumor cells, through the cell-surface glycans, can bind to the glycan-binding receptors - lectins on endothelial cells and immune cells - and thereby affect tumor-cell progression^5^. As an attractive and widely pursued field, this research has attempted to find cancer biomarkers or drug targets in cancer-specific, cell-surface glycans. The majority of studies of glycosylation changes in HCC are conducted using patient serum samples^6^. Few studies have focused on the altered mechanism of the glycoform in tissue samples. Dall'Olio *et al.* demonstrated the dramatic variations of the ST6GAL1 and α2,6-sialylated glycoconjugates when the neoplastic transformation of hepatocytes occurred in 21 HCC cases^7^. Mehta *et al.* found that the tetra-antennary N-linked glycans changed by comparing 16 tumor samples and adjacent tissue^8^.

DNA sequencer-assisted fluorophore-assisted carbohydrate electrophoresis (DSA-FACE), which was developed by Callewaert, has been used to study the profile of serum as a robust, high-throughput, exceedingly sensitive, and reliable quantitative method^9^,^10^. Recently, we utilized this high-throughput glycomics method to profile cell surface-specific N-glycans from human breast cancer cell lines and tissues^11^. In this study, we analyzed 88 pairs of clinical tumor and adjacent tissues and 61 serum samples from 88 HCC patients using this N-glycomics procedure. The results revealed that NA3Fb was highly expressed on the cell surface of HCC cells (*p* < 0.01) and was increased in corresponding HCC patients’ serum samples (*p* < 0.01). Further, we found that the high expression of N-acetylglucosaminyltransferase IVa (GnT-IVa) caused an abnormal increase of NA3Fb on the HCC cell surface. Knockdown of GnT-IVa can lead to potent inhibition of the invasion and migration of HCC cells. This study revealed significant connections between altered N-glycans and the tumorigenesis, progression and metastasis of HCC, indicating GnT-IVa as a therapeutic target for HCC treatment.

## Results

### The profiles and structures of the cell-surface N-glycan of HCC

**Figure 1:**
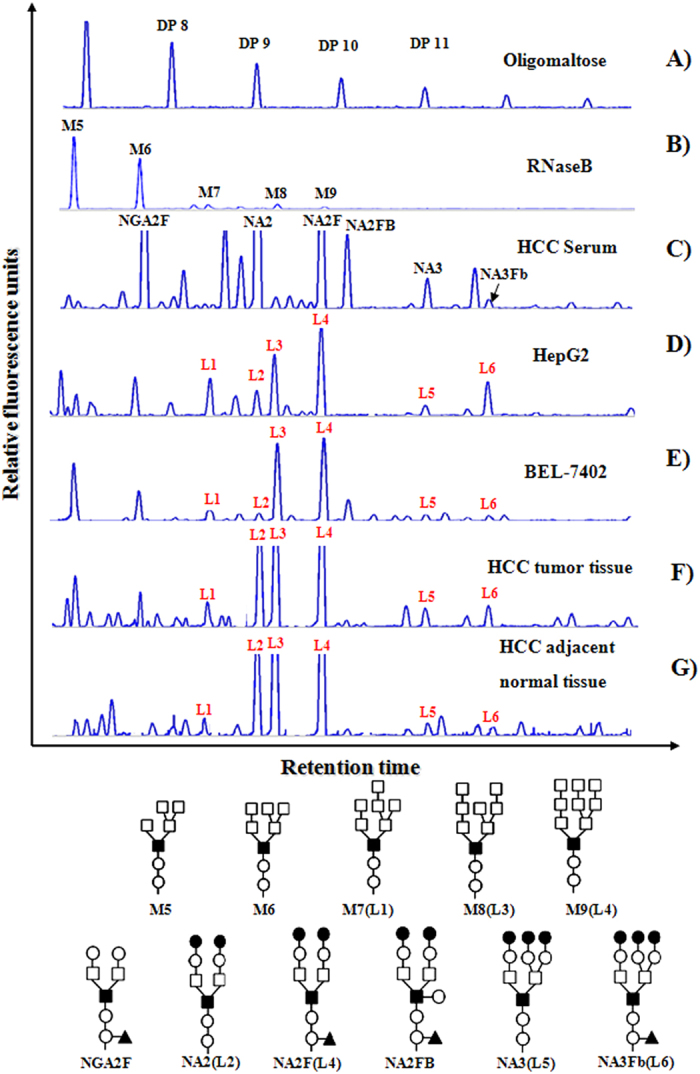
Cell surface N-glycan profiles of cell line and tissue of HCC based on DSA-FACE. (**A**) Oligomaltose is used as a sugar mass reference. The number of glucose units (degree of polymerization, DP) in these structures is indicated. (**B**) N-glycan profiles from RNaseB used as N-glycan profile controls. (**C**) HCC serum contained various complex N-glycans and the most abundant glycans detected in serum are marked. (**D**) and (**E**) are N-glycan profiles of cell surface of two HCC cell line, HepG2 and BEL-7402. (**F**) and (**G**) are N-glycan profiles of cell surface of HCC clinical tissues and adjacent normal tissues. NGA2F is an agalacto core-α-1, 6-fucosylated biantennary glycan; NA2 is a bigalacto biantennary glycan; NA2F is a bigalacto core-α-1, 6-fucosylated biantennary glycan; NA2FB is a bigalacto core-α-1, 6-fucosylated bisecting biantennary glycan; NA3 is tri-antennary; NA3Fb is a core-α-1, 6-fucosylated triantennary glycan. The symbols used in the structural formulas are as follows: (○) β-linked N-acetylglucosamine; (●) β-linked galactose; (□) α-linked mannose; (■) β-linked mannose; (▲) α-1, 6-linked fucose.

DSA-FACE has been proved as a high-throughput tool to analyze cell-surface glycans. We used DSA-FACE to study the profiles and structures of the cell-surface N-glycans of HCC. To determine the structures of the cell-surface N-glycans, we compared their N-glycan profiles with that of RNaseB and HCC serum, whose N-glycan profiles were already known (Fig. 1). Exoglycosidase digestion was used to analyze the structure of the N-glycan pool (Supplementary Figure S1) as Nico Callewaert described in *Nature Medicine*^9^. We assigned the peaks L1 (M7), L2 (NA2), L3 (M8), L4 (M9+NA2F), L5 (NA3) and L6 (NA3Fb) in the cell-surface N-glycan profiles.

### Analysis of the HCC-specific cell-surface N-glycans

In this study, HCC clinical tissues (tumor and adjacent normal tissues, n = 88) were used to study the HCC-specific, cell-surface N-glycans (Table 1). Specimens were characterized according to the patients’ gender, age, tumor grade according to TNM stages and differentiation.

**Figure 2:**
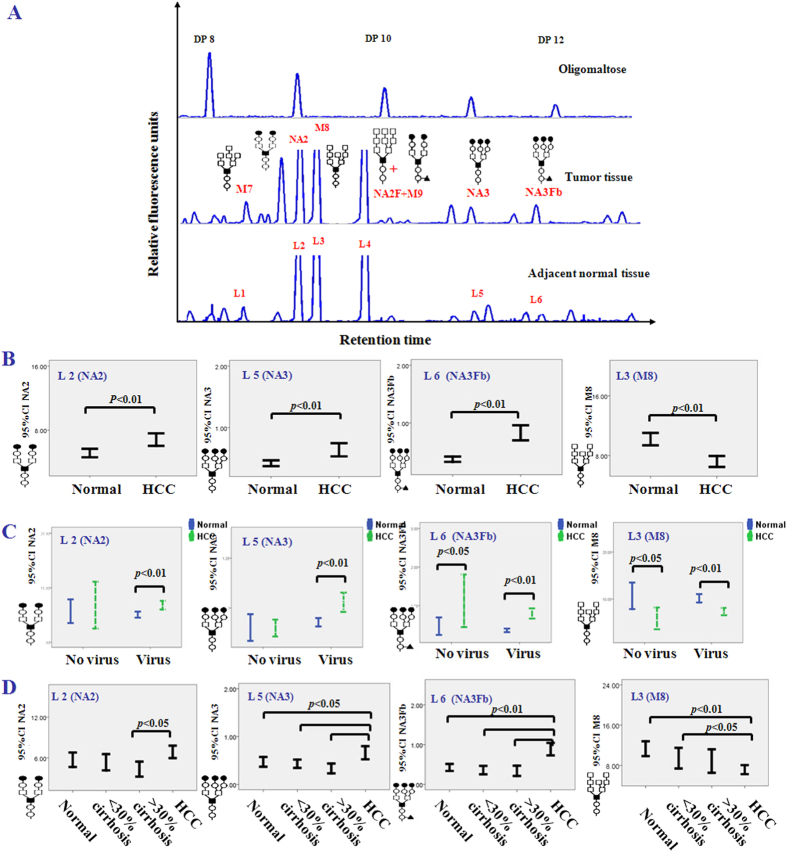
The significantly changed N-glycans in hepatocellular carcinoma tissues compared to adjacent normal tissues. (**A**) The typical cell surface-specific N-glycan profiles from hepatocellular carcinoma tissue and adjacent normal tissue. (**B**) The statistically significant differences of N-glycan peaks NA2, NA3, NA3Fb and M8 between cancer and adjacent normal tissues. (**C**) The relation between the degree of cirrhosis and the expression of NA2, NA3, NA3Fb and M8.

Paired and unpaired *t*-tests were used to compare normal and malignant tissues, and the intra- and inter-assay coefficients of variations (CVs) of the glycan analysis were less than 5%. The results showed that the N-glycan profiles of hepatocellular carcinoma tissues were substantially distinguished from those of the corresponding adjacent tissues (Fig. 2A). The levels of peaks L2 (NA2), L5 (NA3) and L6 (NA3Fb) were significantly increased in the tumor group (*p* < 0.01), but peak L3 (M8) was significantly decreased in the tumor group (*p* < 0.01) compared to the adjacent normal tissue group (Fig. 2B).

Because HCC patients often have viral infectionsand cirrhosis, we further analyzed the relation among the virus, the degree of cirrhosis and the level of these glycans. The results showed that the M8 and NA3Fb have the same tendency between HBV and non HBV patients. NA2 and NA3 were only altered in the group of HCC tissues infected with HBV (Fig. 2C). In addition, based on the degree of cirrhosis, the adjacent tissues were divided into three groups: normal (non-cirrhosis or cirrhosis area <5%), cirrhosisI (5%< cirrhosis area <30%) and cirrhosisII (cirrhosis >30%) for glycan analysis after HE staining and pathological analysis of the frozen tissue section. Typical images of the three groups are shown in Supplementary Figure S2. The results revealed that the level of NA2, NA3 and NA3Fb decreased along with cirrhosis and were increased in HCC (Fig. 2D), which indicated that NA2, NA3 and NA3Fb were not influenced by cirrhosis. The level of M8 decreased with the increased degree of cirrhosis, and its level was the lowest in HCC. Therefore, NA3Fb can be regarded as a specific glycan on the surface of HCC.

The concentrations of a biantennary glycan (NA2), triantennary glycans (NA3 and NA3Fb) and a high-mannose glycan (M8) in hepatocellular carcinoma patients with different gender, age, clinical stage and tumor differentiation degree were compared to study the correlation between these N-glycans and the clinical characteristics. Peak L6 (NA3Fb) was significantly different in male and female patients, with age < = 50 and age >50 and in different TNM stage groups (Supplementary Figure S3) (*p* < 0.01). Based on this observation, we speculated that peak L6 (NA3Fb) is associated with the malignant progression of hepatocellular carcinoma.

### The change of NA3Fb in serum

**Figure 3:**
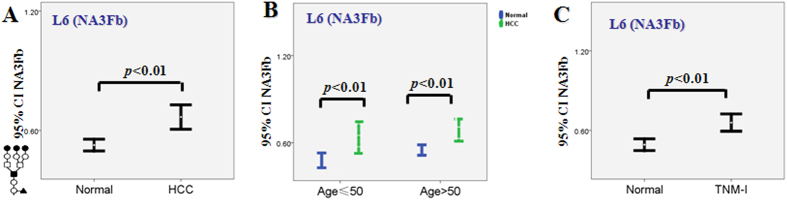
Analysis of the alternation of NA3Fb in the serum and tumor cells of same patient. The changes of NA3Fb in clinical tissues and serum of 61 HCC patients (**A**), different ages (**B**) and at TNM-I stage (**C**).

Based on the results of the specific cell-surface glycan of HCC tissue, we continued to study the changes of NA3Fb in serum. The serum specimens of 61 from 88 HCC patients (Supplementary Table S3),along with 90 age- and gender-matched healthy humans as a control group, were analyzed using DSA-FACE. The results showed that the NA3Fb tendency is the same in clinical tissues and serum. The level of NA3Fb was significantly increased in the tumor group (*p* < 0.01) in serum (Fig. 3A). In addition, the level of NA3Fb was also increased in different age groups, indicating that it is age-independent (Fig. 3B). We analyzed only TNM-I stage patients in this study. The serum result was consistent with the clinic tissue, showing that the level of NA3Fb was higher in cancer group than in the control group (Fig. 3C), which further proved that the higher level of NA3Fb is caused by the changes of the synthetic pathway and has close relation with HCC.

### The increase of NA3Fb in the cell surface accompanied by the high expression of GnT-IVa

**Figure 4:**
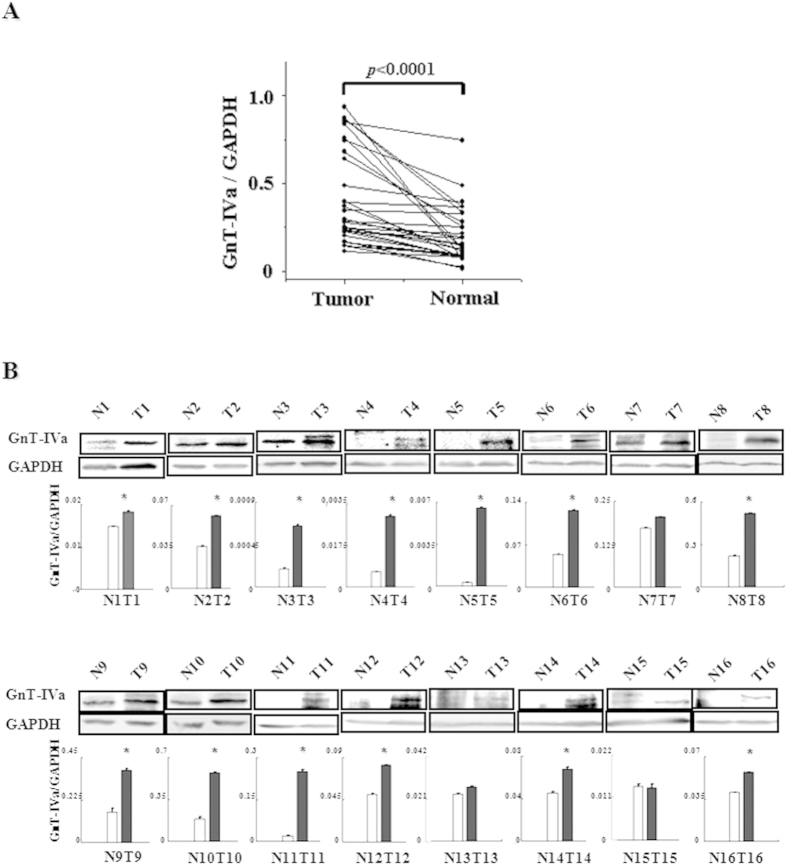
The expression of *MGAT4A* correlation with the structure of NA3Fb. (**A**) The relative expressions of *MGAT4A* was determined by RT-PCR from 34 pairs of HCC and adjacent normal tissues. Statistical analysis was performed comparing HCCs with normal surrounding tissues. (**B**) Western blot analysis of GnT-IVa expression in 16 pairs of HCC and adjacent normal tissues. The results were normalized to the amount of GAPDH. *means *P* < 0.05.

After revealing the increase of NA3Fb in HCC clinical tissue and serum, we attempted to further investigate the related molecular mechanism. Because these glycan structures are synthesized by a series of glycosyltransferases, the transcriptional expression levels of 5 glycosyltransferases related with NA3Fb were studied,including *FUT8* (fucosyltransferase 8, Fut8), *GALT-1*(galactose-1-phosphate uridylyltransferase, GalT-1), *MGAT5* (N-acetylglucosaminyltransferase V, GnT-V), *MGAT4A* and *MG*AT4B (N-acetylglucosaminyltransferase IVa and IVb, GnT-IVa and GnT-IVb). Thirty-four pairs of HCC tissue and adjacent normal tissue were used to study the 5 gene expressions by qRT-PCR. Among the 5 glycosyltransferases, only GnT-IVa expression was substantially increased in HCC tissues based on the pairwise comparison between individual HCC and paired adjacent normal tissue (*p* < 0.01) (Fig. 4A); the results of the other 4 glycosyltransferases are shown in Supplementary Figure S4.

To confirm the results of the qRT-PCR, the protein expression of GnT-IVa was evaluated in 16 HCC tissues and their adjacent normal tissue (Fig. 4B). The results showed that in 13 of 16 cases, the expression of GnT-IVa was increased in HCC tissues compared to the adjacent normal tissue, whereas in the other 3 cases, the expression level was similar in both tissues. These protein expression results are consistent with those of the transcriptional expression. Collectively, these findings indicated that the high expression of GnT-IVa has close relation with the high level of NA3Fb.

**Figure 5:**
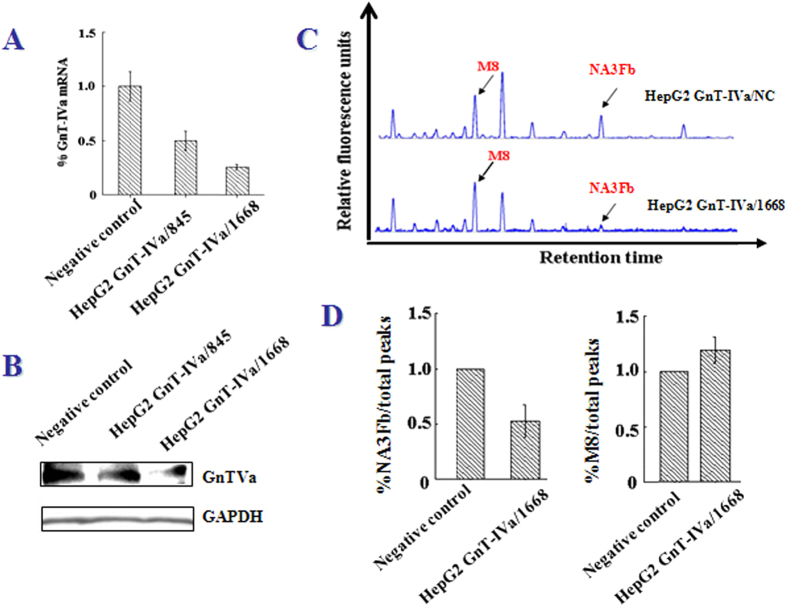
The relative quantity of HepG2 cell surface NA3Fb at 48 h after the transfection of GnT-IVa. (**A**) qRT-PCR and (**B**) Western blot analysis of GnT-IVa expression. (**C**) The DSA-FACE figure of the cell surface glycan. (**D**) The normalization results of intensity of NA3Fb and M8.

**Figure 6:**
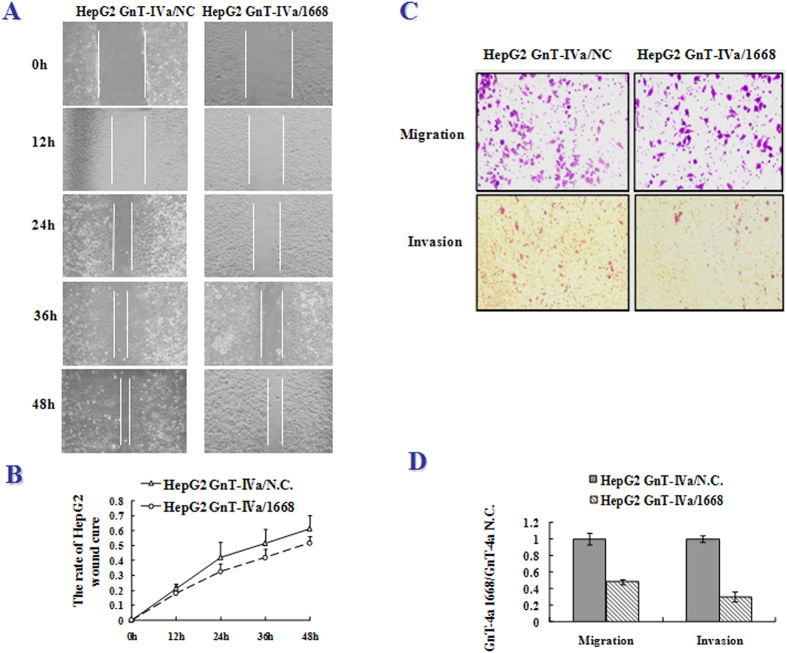
The GnT-IVa is related with cell migration of HepG2. (**A**) The picture of scratch wound migration assay. Healing due to cell migration of HepG2 was observed over a period of 6 h and 48 h following scratch wounding. (**B**) Quantitative data from the cell scratch healing assay is shown. (**C**) The picture of cell migration and invasion using a Transwell assay. (**D**) Quantitative data of cell migration and invasion.

Subsequently, GnT-IVa was knocked down in HepG2 cells by transfection with GnT-IVa shRNA (Fig. 5A,B). The results revealed that the level of NA3Fb decreased and M8 increased in knockdown GnT-IVa/1668 cells compared with GnT-IVa/NC after 48 h transfection (Fig. 5C,D). In addition, analysis of cell scratch healing and transwell migration in vitro was used to study the alteration of cell migration. The healing due to cell migration of HepG2 was observed over periods of 6 h and 48 h following scratch wounding (Fig. 6A). The quantitative data showed that the healing rates of the GnT-IVa/1668 group decreased more than the GnT-IVa/NC group (Fig. 6B). HepG2/1668, and HepG2/NC cells were seeded into the upper chamber of a transwell insert(Fig. 6C). The transwell migration and invasion test indicated that the number of cells invading the transwell of GnT-IVa/1668 was substantially reduced in comparison with GnT-IVa/NC (Fig. 6D). Similar results were obtained in another HCC cell line-BEL-7402(Supplementary Figure S5).

These results proved that the high expression of GnT-IVa leads to a higher level of NA3Fb in the HCC cell surface, and the downregulation of GnT-IVa may modulatecell invasion and migration through NA3Fb.

## Discussion

As the third biomacromolecules, glycans are becoming increasingly important in biological, chemical and clinical research and applications. Abnormal changes of glycans in both structures and amounts are closely related to many diseases. Altered glycosylation is a universal feature of many types of cancers, including HCC^12^. However, more studies focus on serum and serum biomarkers.

DNA sequencer-assisted fluorophore-assisted carbohydrate electrophoresis (DSA-FACE) is a useful high-throughput method for N-glycome profiling. This technique has been successfully applied for screening serum glycoprotein biomarkers from healthy people^13^,^14^, patients suffering various diseases^15^,^16^,^17^,^18^,^19^ and cancer patients^20^,^21^,^22^,^23^,^24^,^25^,^26^,^27^. Our group previously studied the relation of serum glycan and aging using DSA-FACE^28^. Recently, we modified the DSA-FACE technique and used it to profile the cell surface-specific N-glycans in breast cancer^11^. In this study, we used DSA-FACE to analyze the N-linked glycan profiles of clinical HCC patient tissue and serum to identify the specific N-glycans of the cell surface in HCC and the molecular mechanism of their biosynthesis.

In our study, the profiles of the cell-surface N-glycans of HCC and 6 typical glycan structures were revealed. NA2 and NA3 were significantly increased in the tumor group compared to the adjacent normal tissue group. NA2 is a bigalacto-biantennary and NA3 is a tri-antennary glycan. Several previous studies showed NA2 and NA3 as serum markers in different diseases of the liver. Goldman *et al.* found that the NA2 was less abundant in hepatocellular cancer (HCC) patients with hepatitis C virus (HCV) than in healthy humans^12^. Chen *et al.* found that NA2 was lower in a nonalcoholic steatohepatitis (NASH) group than in simple fatty liver (NAFL) and control groups^17^. Callewaert *et al.* found that during liver fibrosis progression, the amount of NA3 concentration decreases in the serum *N*-glycome^18^. Gui *et al.* showed that the biantennary (NA2) and the triantennary (NA3) N-glycans decreased significantly with increased severity of fibrosis^19^. These results showed that NA2 and NA3 in serum were decreased in more serious stages of liver disease, which is similar to our results showing that NA2 and NA3 decreased in 61 HCC serum samples (Supplementary Figure S6). However, our results showed that the levels of NA2 and NA3 were increased in HCC tissues compared to adjacent normal tissue in the virus group. There is an opposite variation tendency of NA2 and NA3 inthe serum and tumor tissue of the same patientdue to the different secreting pathways of secreted and resident glycoproteins. The abundance of NA2 and NA3 in HCC tissue with virus indicates that they can be used as therapeutic targets for cancer treatment. Riproximin, a new type II ribosome-inactivating protein with antineoplastic activity from *Ximenia americana*, preferentially binds to the branched bi- and triantennary N-glycan structures NA2 and NA3^29^, which may help riproximin to recognize the glycans in cancer cells, resulting in tumor-specific cytotoxicity.

In addition, the level of NA3Fb, a core-α-1, 6-fucosylated triantennary glycan, increased not only on the cell surface of HCC cells but also in corresponding HCC patients’ serum samples. The abnormal increase of NA3Fb appeared at the early HCC stages and was independent of patients’ age. Nakagawa demonstrated that this glycan was increased in α-fetoproteins (AFPs) from HCC by detecting the N-glycan structures of AFPs from cell lines, serum and ascites fluids from HCC patients^30^. A similar core, fucosylated, tri-antennary glycan with sialic acid, G2890, was increased in the serum of the HCC group and was viewed as a promising biomarker for malignant behavior in HCCs^31^. Our previous studies showed that this glycan was increased in breast cancer serum and tissue^11^. Cancer cells frequently display glycans at different levels or with fundamentally different structures than those observed in normal cells^32^. The glycans have been proven to be in direct relation with cancer oncogenesis, and some have been regarded as tumor-associated antigens, such as mucin-related Tn, Sialyl Tn, and the blood group Lewis-related Lewis Y and Sialyl Lewis X^33^. Therefore, the alteration of the core-α-1, 6-fucosylated triantennary glycan in the serum and tissue of breast cancer and HCC may reflect the change of the molecular mechanism in cancer oncogenesis and may serve as a new tumor-associated antigen or drug target.

Glycosylation is controlled by both glycosyltransferases and glycosidases and responds to the cellular microenvironment as a result of cellular responses^34^. The biosynthesis of complex N-glycans is catalyzed by the action of N-acetylglucosaminyltransferases (GnTs) in the medial-Golgi apparatus, which adds antennae branching structures to the common core structure. GnT-IV and GnT-V are required for the formation of tri- and multiantennary branching structures on membrane proteins that require glycosylation as a signal for exportation in the Golgi apparatus^35^. Therefore, GnT-IV may have functional roles that are no less important than those of GnT-V. GnT-IVa and GnT-IVb isoenzymes have GnT-IV enzymatic activity and share substrate specificity with different tissue distribution profiles^36^. In this study, we further investigated the molecular mechanism of the considerable increase of NA3Fb in HCC patients. The results indicated that both the mRNA and protein expression of GnT-IVa were substantially increased in HCC tissues compared with the corresponding adjacent tissues. Knockdown of *mgat4a* in HepG2 cells revealed that the decrease of NA3Fb was directly related with the gene silencing of GnT-IVa. GnT-IV can transfer an N-acetylglucosamine (GlcNAc) group to the core α 1–3 mannose of N-glycans, forming a β 1–4 linkage, and can act on biantennary sugar chains and generate triantennary sugar chains^37^. The distinct N-glycan branch catalyzed by GnT-IV is the common substrate for GnT-V, with a ß1,4- or ß1,6-linked N-acetylglucosamine residue^38^. Although GnT-IV and GnT-V (the research hot spot of the GnT family) have similar function and are required for the formation of multiantennary, complex-type N-glycans, there are few studies on GnT-IV compared with GnT-V.

GnT-IVa has a strong relation with many diseases, including type 2 diabetes^39^, choriocarcinoma^40^, pancreatic cancer^41^,and primary colorectal carcinoma^42^. However, there are few studies investigating the relationship between GnT-IVa expression and hepatocarcinoma. Deguchi *et al.* showed that the GnT V-catalyzed N-glycan products did not increase, but a clear increase in the proportion of GnT IV products was observed in mouse tumors^43^. This is in agreement with our results showing that the expression of GnT-IVa substantially increased in HCC tissues and the products of the GnT-Iva-catalyzed, core-α-1, 6-fucosylated triantennary glycan increased on the cell surface and in the serum of the same HCC patients. Further studies showed that GnT-IVa regulatesthe migration and invasion of HCC cells, which is similar to Fan’s research^44^. Mice deficient in GnT-IVa demonstrated that GnT-IVa is essential for the production of a complex-type glycan ligand on pancreatic β cells^45^, which indicated that GnT-IVa may play important roles during the process of hepatocarcinoma growth and metastasis. These studies demonstrated that the formation of proper antennal complexity on N-glycans is crucial for maintaining biological processes in normal contexts^34^. Our preliminary study of this abnormal glycosylation mechanism provided new paths for the research of the carcinogenesis and malignant progression of HCC.

Moreover, we found that M8 was significantly decreased in the tumor group (*p* < 0.01) compared with the adjacent normal tissue group. M8 was increased by approximately 20% in the GnT-IVa knockdown HepG2 cell lines by mgat4a-homo-1668. The high-mannose-type N-glycans play an important role in allowing only properly folded proteins to pass the quality control system in the endoplasmic reticulum and to move to the Golgi apparatus. The N-glycans are subsequently processed by Golgi-resident glycosyltransferases and glycosidases and acquire complex branch structures bearing a variety of glycan moieties^46^. The high mannose M8 is the precursor and intermediate product of N-glycan synthesis. The increasing level of NA3Fb and the decreasing level of M8 may be explained by the increased activity of GnT-IVa. Most of the M8 was used as a substrate to synthesize NA3Fb, which led to an increased abundance of NA3Fb, and minimal M8 was left during the cell transformation.

In conclusion, the current study revealed the HCC tissue N-glycan profiles and the specific glycans on the HCC cell surface: NA2, NA3, M8 and NA3Fb. The change of NA3Fb was not influenced by HBV or cirrhosis. NA3Fb can be regarded as a potential drug target for tumor therapy. Furthermore, the mechanism study showed that the increase of NA3Fb results from the increased expression of GnT-IVa. GnT-IVa regulates cell migration and invasion through NA3Fb and may play important roles in HCC. This study provided additional insight and led to a better understanding of the mechanisms of tumorigenesis, progression and metastasis of HCC by confirming that altered glycosylation is involved in the pathogenesis of malignancies.

## Methods

### Tissue and serum specimens

The Tumor Hospital of Harbin Medical University provided 88 pairs of matched adjacent normal and tumor specimens and 61 serum specimens from 88 patients with histologically proven primary hepatocarcinoma. 90 age- and gender-matched serum specimens from healthy human were used as serum control group. The stage was classified according to the TNM criteria (2002)^47^. The research was approved by the Ethical Committee of the Tumor Hospital of Harbin Medical University, and was in accordance with the ethical standards laid down in the 1964 Declaration of Helsinki. The written informed consents of all participating subjects were obtained before commencement of the study.

### N-glycome analysis of cell membrane and serum

The N-glycans analysis of cell membrane was performed as described previously^11^. In brief, the ultracentrifugation was used to separate the cell membrane fractions. The N-glycans present on the cell membrane proteins were released by PNGase F (NEB). Sialic acid was removed with neuraminidase (NEB). The N-glycans were labeled with 8-amino-1, 3, 6-pyrenetrisulfonic acid (APTS, Invitrogen) and purified with Sephadex G10 (Invitrogen). Finally, the samples above were analyzed by ABI3130 Genetic Analyzer (Applied Biosystems). The results were analyzed using the GeneMapper 4.0 software (Applied Biosystems). Each N-glycan was numerically described by normalizing its height to the sum of the heights of all peaks. Serum N-glycome analysis was performed as described previously^28^. The exoglycosidase digestion was used to analyze the structure of N-glycan pool^9^.

### HE Staining

8 μm-thick sections of frozen tissues were cut and placed on glass slides. Tissue samples were stained with hematoxylin and eosin.

### Cell culture and shRNA transfection

The human HepG2 and BEL-7402 cell were obtained from ATCC and the Shanghai Institute Cell Bank, respectively. For glycan analysis, cells were detached using a cell scraper, washed with PBS, pelleted and frozen at −80 °C until analysis. GnT-IVa and scrambled shRNA were purchased from Ji Ma Company (China). The shRNA sequences targeting *mgat4a* were showed in Supplementary Table S2. The shRNAs were transfected using Lipofectamine^TM^ 2000 (Invitrogen, USA) and then incubated for 24 h to detect the mRNA and 48 h to analyze the GnT-IVa protein level.

### RT-PCR and Western Blot

Methods of RNA extraction and real-time RT-PCR analysis were conducted as previously described^48^. The primers used in PCR were showed in Supplementary Table S1. The total protein was gathered and the GnT-IVa antibodies (Abcam company) and GAPDH (Santa Cruz Biotechnology) were used in western blot process. And the band intensity was quantified by arithmetic analysis using the software Image Studio.

### The Wound Healing and Transwell

The wound healing and transwell test were conducted as previously described^49^. They were used to study the function of the GnT-IVa by comparing the RNA interference of HCC cell lines and negative control.

### Statistical analysis

Data from at least three independent experiments were used to compare between groups. All quantitative variables were presented as mean ± SD. Paired and unpaired *t*-tests were used for comparison of the two tissue groups and serum specimens. Student’s *t*-test was used to determine statistical significance for the intergroup comparisons. Statistical analysis was performed with SPSS 10.0 software.

## Additional Information

**How to cite this article**: Nie, H. *et al.* Specific N-glycans of Hepatocellular Carcinoma Cell Surface and the Abnormal Increase of Core-α-1, 6-fucosylated Triantennary Glycan via N-acetylglucosaminyltransferases-IVa Regulation. *Sci. Rep.* **5**, 16007; doi: 10.1038/srep16007 (2015).

## Supplementary Material

Supplementary Information

## Figures and Tables

**Table 1:** Clinical Characteristics of the Study Population^a^ Located on page 7.

	Characteristics	Category	Cases	Percentage (%)
Paired tissues (n = 88)	Gender	Male	73	83.0
		Female	15	17.0
	TNM stage	I	68	77.3
		II	9	10.2
		III^a^	5	5.7
		III b	1	1.1
		ND	5	5.7
	Grade of differentiation	G1	4	4.5
		G2	16	18.2
		G3	31	35.2
		ND	37	42.1
	Degree of cirrhosis	<5%	34	38.6
		5%–30%	19	21.6
		>30%	9	10.3
		ND	26	29.5
	Virus	virus	80	90.9
		Non-virus	8	9.1
	Age	<40	7	8.0
		41–50	30	34.0
		51–60	38	43.2
		>60	13	14.8

^a^ND, not determined; G1, high grade; G2, moderate grade; G3, poor grade.
