# Supplementary material for: Specific N-glycans of Hepatocellular Carcinoma Cell Surface and the Abnormal Increase of Core-α-1, 6-fucosylated Triantennary Glycan via N-acetylglucosaminyltransferases-IVa Regulation
Source: Sci Rep. 2015 Nov 5;5:16007. doi: 10.1038/srep16007 (PMC4633583; doi:10.1038/srep16007)
Supplement: Supplementary Information [file srep16007-s1.doc]

**Specific N-glycans of Hepatocellular Carcinoma Cell Surface and the Abnormal Increase of Core-α-1, 6-fucosylated Triantennary Glycan via N-acetylglucosaminyltransferases-IVa Regluation**

**Huan Nie^1§^, Xia Liu^1,4§^, Yubao Zhang^3§^,** Tingting Li^1^, Chao Zhan^3^, Wenjuan Huo^1^, Anshun He^1^, Yuanfei Yao^1^, Yu Jin^3^, Youpeng Qu^1^, Xue-Long Sun^2^ and Yu Li^1*^

Fig.S1


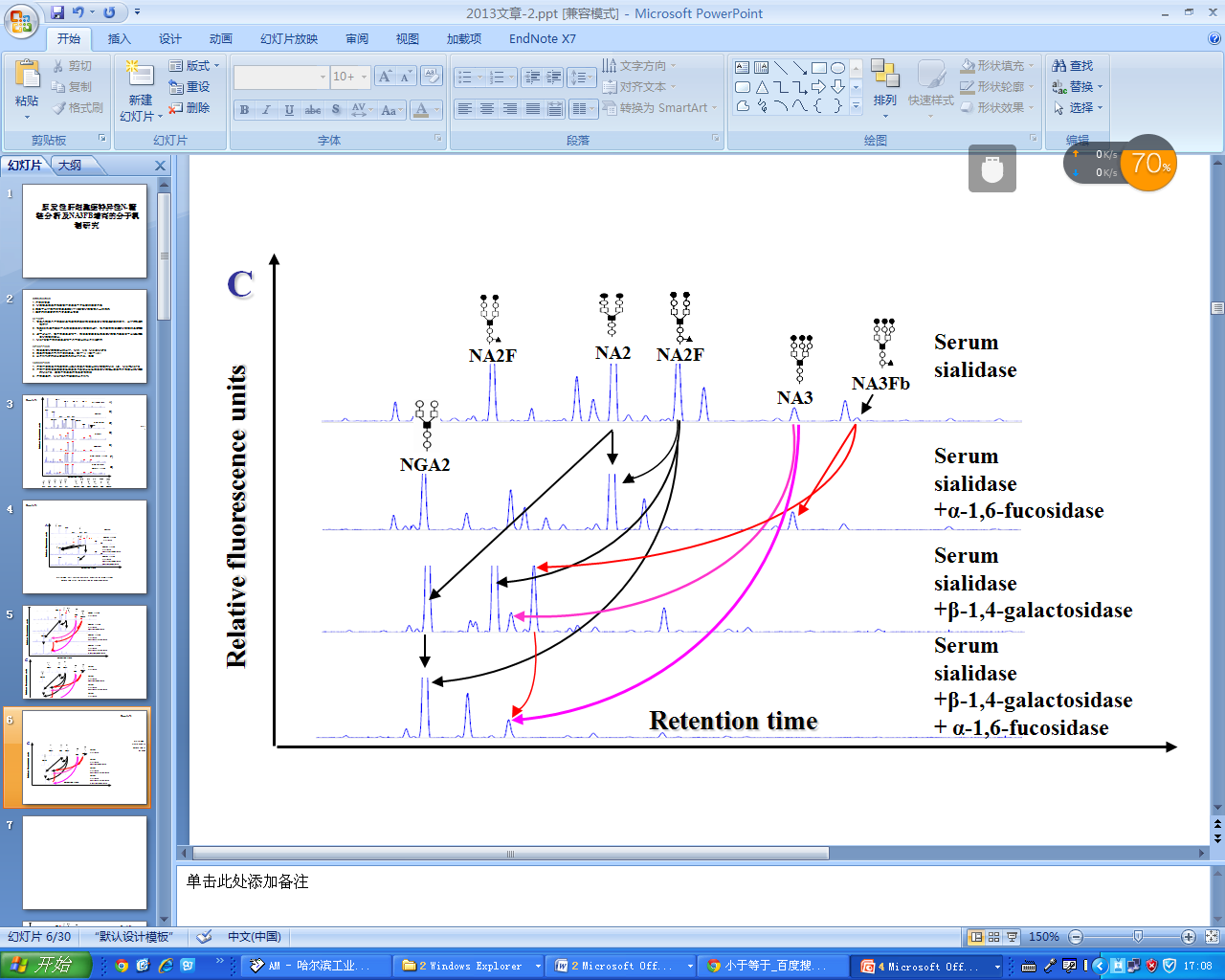

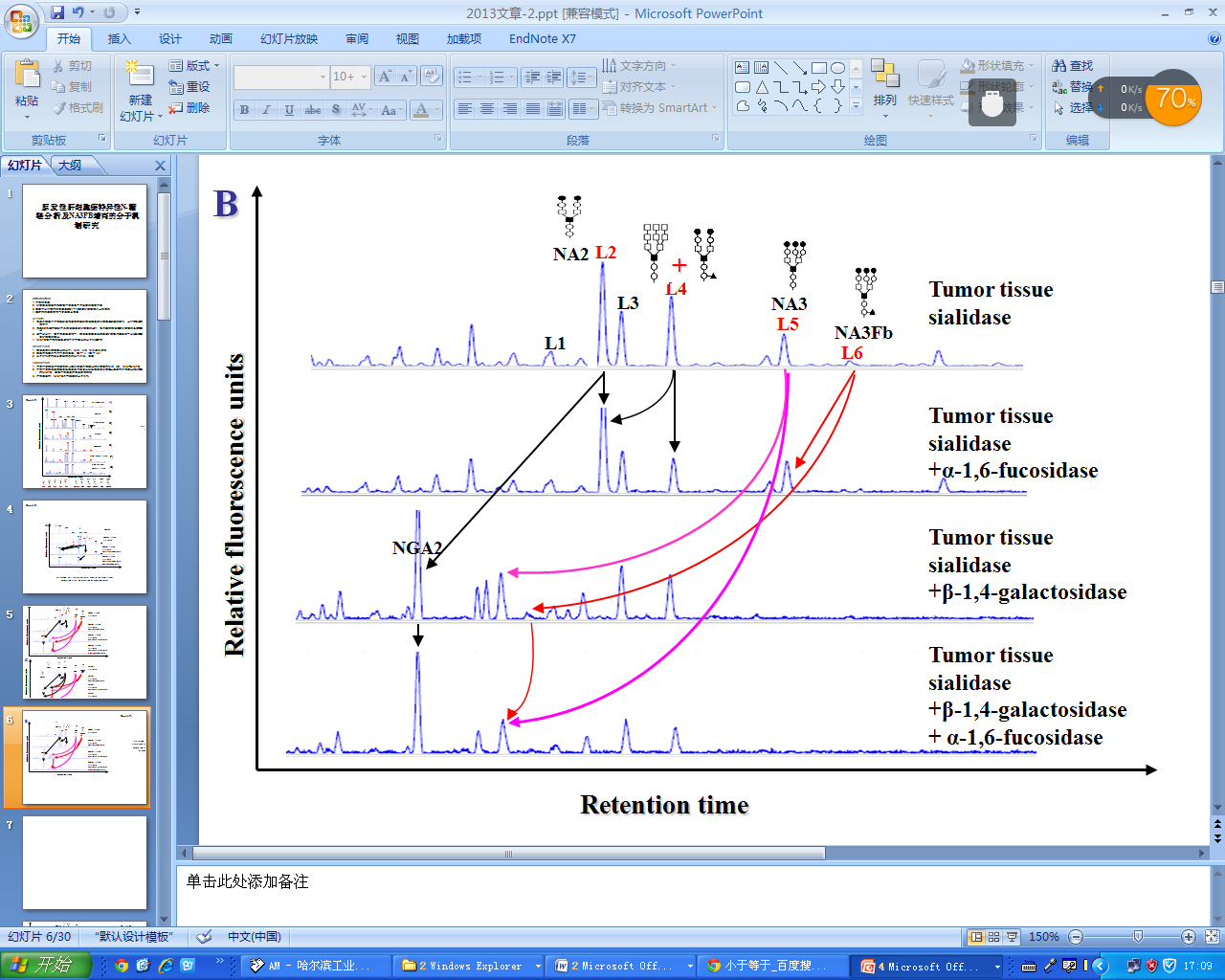

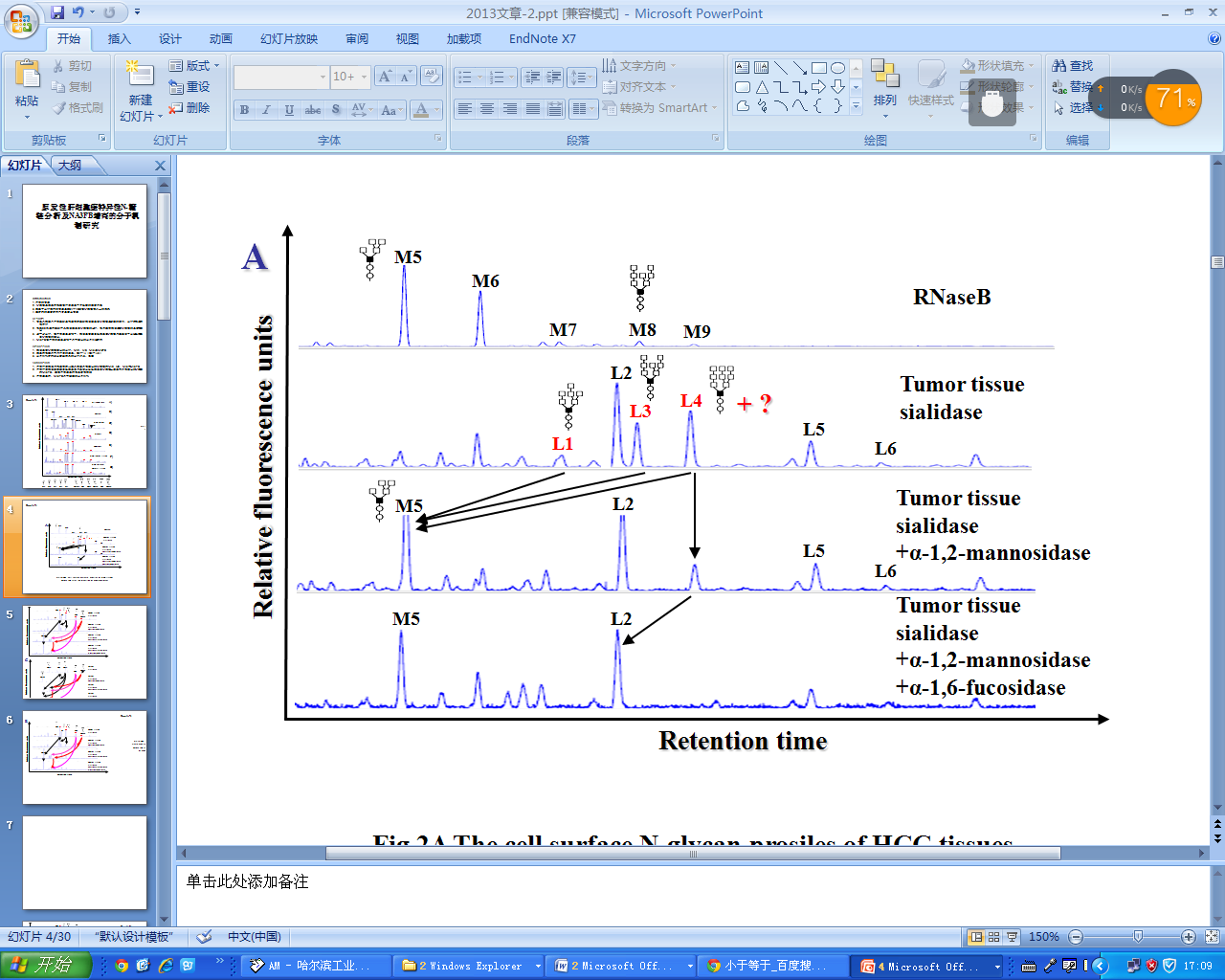


**Fig.S1**  The exoglycosidase sequencing of the cell surface N-glycans from tumor tissues. (A) The total N-glycans were treated with α-1, 2-mannosidase or combined α-1, 6-fucosidase to verify the structures of peaks L1, L3 and partial L4. (B) Exoglycosidase sequencing of cell surface N-glycans from HCC tissue and glycoproteins from human serum as reference (C) to verify the structures of peaks L2, L4, L5 and L6. The total N-glycans were treated with single or combined exoglycosidase arrays as indicated in context. The arrow lines indicate the changes in glycan peaks that underwent glycosidase digestion. The nomenclature of N-glycans and symbolic representations correspond to those in Figure 1.

In order to determine the structures of the cell surface N-glycans, we compared their N-glycan profiles with that of RNaseB and HCC serum whose N-glycan profiles were already known (Figure 1). Peaks L1, L3 and L4 migrated at the same rates as M7, M8 and M9 or NA2F; peaks L2, L5 and L6 migrated at the same rates as NA2, NA3 and NA3Fb, respectively, in the corresponding RNaseB and human serum N-glycan profile (Figure 1). Furthmore, the N-glycan structures of peaks L1, L3 and L4 were digested by α-1, 2-mannosidase. The results showed that peaks L1 and L3 disappeared and generated M5, confirming peaks L1 and L3 represented high mannose 7 (M7) and 8 (M8) respectively (Figure S1A). However, peak L4 diminished partially and shifted to M5. Following digestion with bovine kidney α-1, 6-fucosidase which can remove only α-1, 6-fucose from the core N-acetylglucosamine, the remained part of L4 disappeared then and shifted one residue forward to L2, indicating L4 contained a mixture of M9 and core-fucosylated N-glycan (Figure S1B). The N-glycan structures of peaks L2, L5 and L6 were further studied by exoglycosidase digestion (Figure S1B). Two, two and three galactoses could be removed from N-glycans responding to peaks L2, L5 and L6, respectively, after β-1,4-galactosidase digestion. When β-1, 4-galactosidase was combined with α-1, 6-fucosidase, one extra fucose residue was taken off from peak L6, suggesting peak L6 was the substrates of α-1,6-fucosidase. These results indicated that peak L6 was a core-α-1,6-fucosylated triantennary glycan (NA3Fb). Moreover, the motion trails of peaks L2 and L5 that had undergone the digestion of β-1, 4-galactosidase or α-1, 6-fucosidase, or their mixture, were consistent with those of the reference glycans NA2 and NA3 in human serum (Figure S1C). Thus, the structures of peaks L2 and L5 were confirmed as NA2 and NA3, respectively. Overall, we assigned the peaks L1, L2, L3, L4, L5 and L6 in the cell surface N-glycan profiles from HCC as M7, NA2, M8, (M9+NA2F), NA3 and NA3Fb, respectively.

Fig.S2


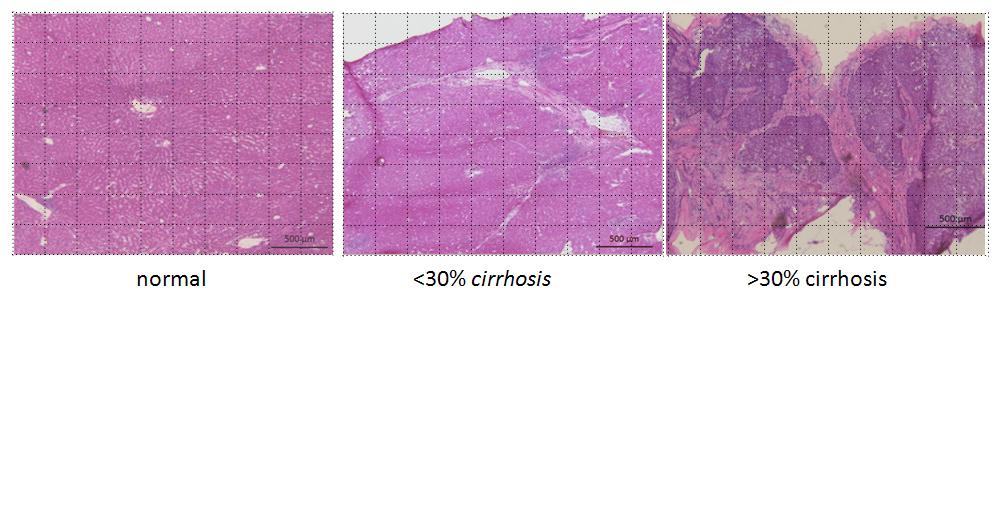


**Fig.S2** The degree of the cirrhosis in adjacent tissues. The adjacent tissues were divided into three groups: normal (cirrhosis area <5%), 5< cirrhosis area<30% and >30% after HE staining and pathological analysis of the tissue frozen section.

Fig.S3


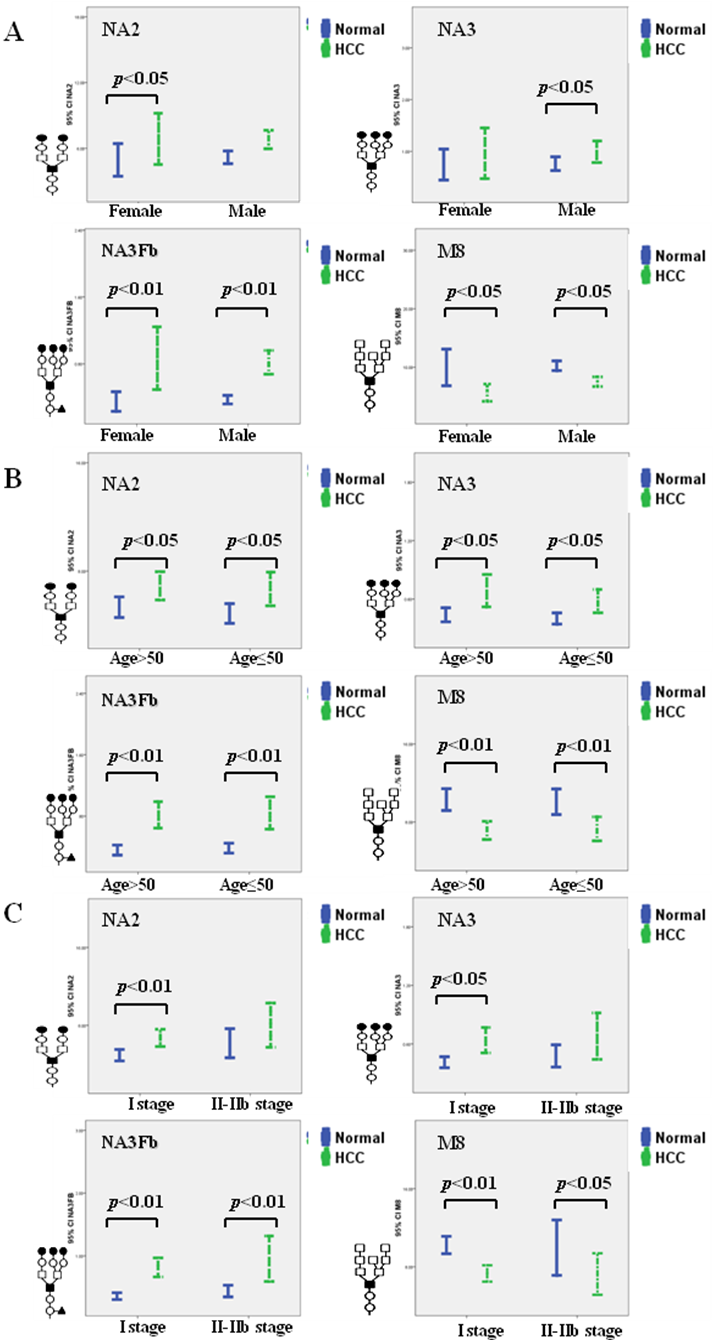


**Fig.S3** The significantly changed N-glycans in hepatocellular carcinoma tissues according to gender, age, clinical stage and grades of differerntiation. (A) The correlation of cell surface specific N-glycans with male and female patients. (B) The correlation of cell surface specific N-glycans with two age groups. (C) The correlation of cell-surface specific N-glycans with clinical stages. Statistically significant differences between groups are indicated by *p* value.

Fig.S4


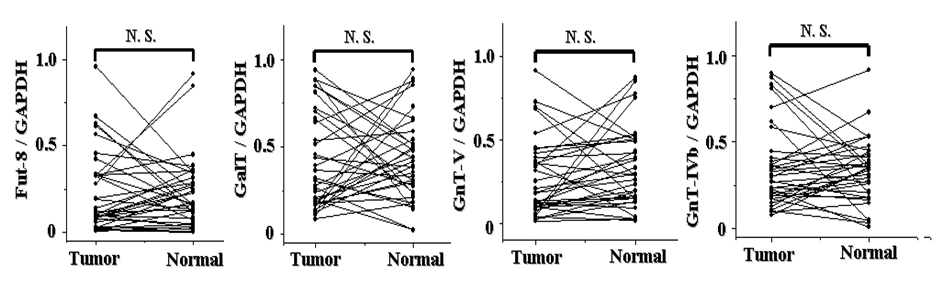


**Fig.S4** The expression of glycosyltransferase correlation with the structure of NA3Fb. The relative expressions of FuT-8, GalT, GnTV and GnTIVb were determined by real-time reverse transcription-polymerase chain reaction (RT-PCR) from 34 pairs of HCC and adjacent normal tissues. Statistical analysis was performed comparing HCCs with normal surrounding tissues.

Fig.S5


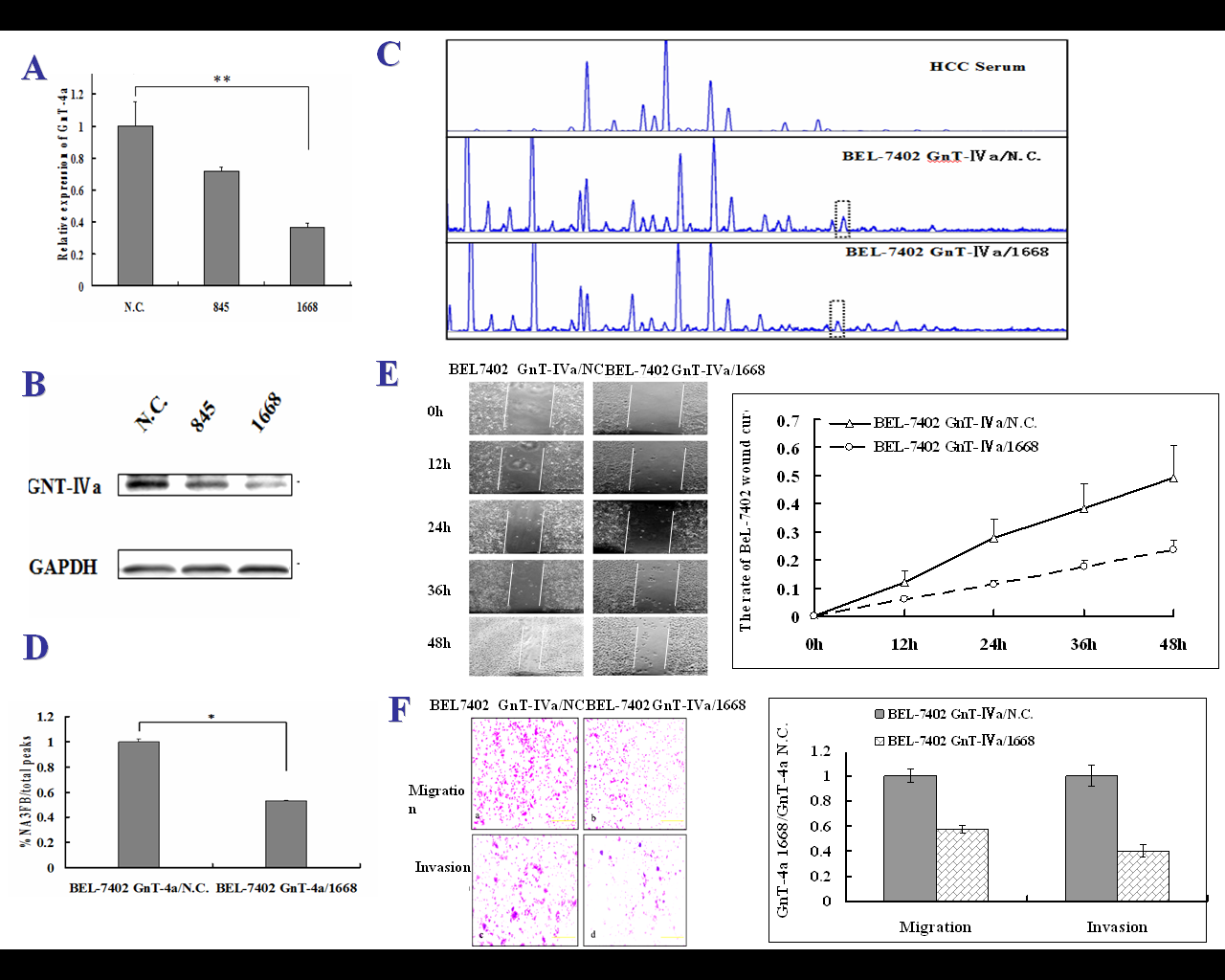


**Fig.S5** The relative quantity of BEL-7402 cell surface NA3Fb at 48h after the transfection of GnT-IVa. qRT-PCR (A) and (B) Western blot analysis of GnT-IVa expression. (C) The DSA-FACE figure of the cell surface glycan. (D) The normalization results of intensity of NA3Fb. (E) Left, the picture of scratch wound migration assay. Healing due to cell migration was observed over a period of 6h and 48h following scratch wounding. Right, quantitative data from the cell scratch healing assay is shown. (F) Cell migration and invasion were measured using a Transwell migration assay. Left, the picture of Transwell insert. Right, quantitative data on cell migration and invasion are shown.

Fig.S6


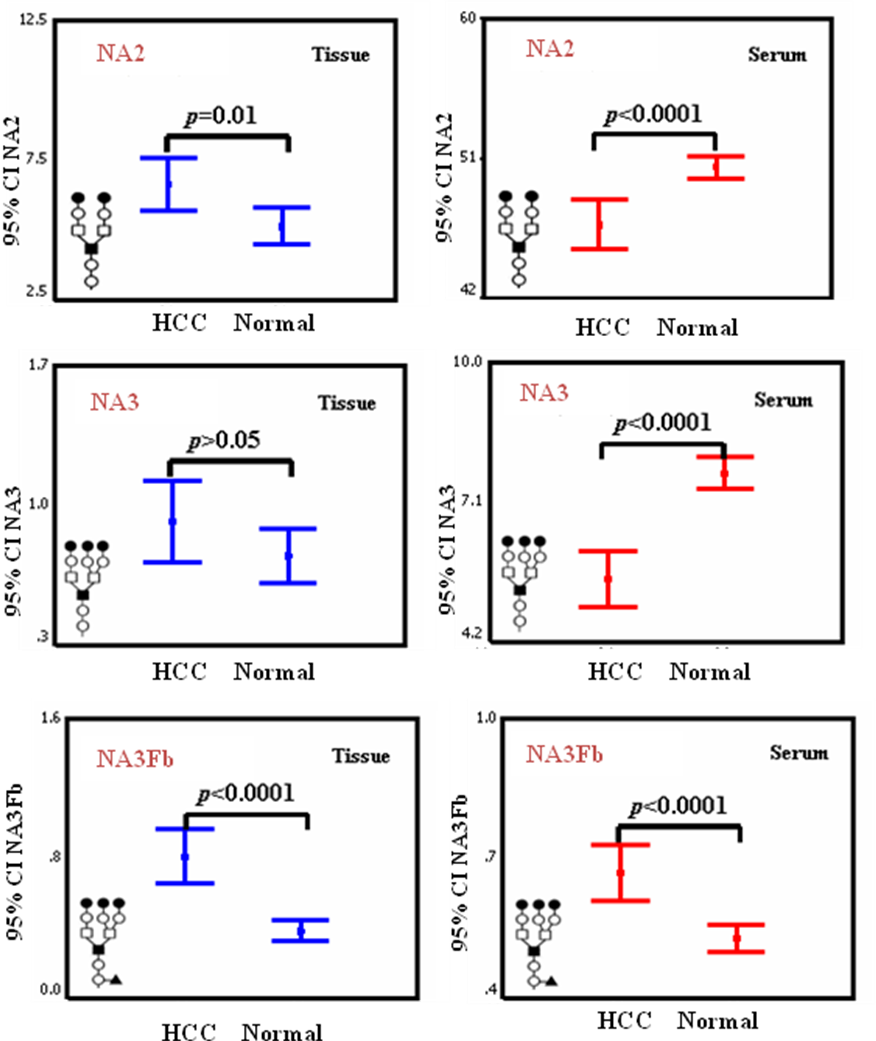


**Fig.S6** Analysis of the alternation of NA2 and NA3 in the serum and tumor tissue of same patient.

**Table S1. The primers of glycosyltransfases used in real-time RT-PCR**

| **Gene name** | **Primer sequence** |
| --- | --- |
| *GnT-IVa* | Sense: 5′- GGCTCCGCAATGGAACTGTA -3′  Antisense: 5′- GCAAGGAATTCTCGTTGATAAGCA -3´ |
| *GnT-IVb* | Sense: 5′- ACTTCATCCGCTTCCGCTTC -3′  Antisense: 5′- TCCTTGTCTGACTGAGGGTTGT -3´ |
| *GnT-V* | Sense: 5′- GAGCAGATCCTGGACCTCAG -3′  Antisense: 5′- GCTGTCATGACTCCAGCGTA -3´ |
| *Fut8* | Sense: 5′- CAGACAGATGGAGCAGGTGA -3′  Antisense: 5′- ACCACATGATGGAGCTGACA -3´ |
| *β-1,4-GalT-1* | Sense: 5′- CGTTGCAATGGATAAGTTTGGATTC -3′  Antisense: 5′- CAGCATTTGGGCGAGATATAGACA -3´ |
| *GAPDH* | Sense: 5′- AACAGCCTCAAGATCATCAGC -3′  Antisense: 5′- GGATGATGTTCTGGAGAGCC -3´ |

**Table S2. shRNAs sequence designed based on the *mgat4a***

| **Name** | **Sequence** |
| --- | --- |
| mgat4a-homo-845, | 5′- GAGAGACAGAUAUUGAUUATT -3′ |
| mgat4a-homo-1668, | 5′- CCUGGAGAUAUUCUGCUAATT -3′ |
| Scrambled siRNA (NC) | 5′- UUCUCCGAACGUGUCACGUTT -3′. |

**Table S3. Clinical Characteristics of the HCC and health population used for**

**serum N-glycan profiles**

|  | Characteristics | Category | Cases | Percentage (%) |
| --- | --- | --- | --- | --- |
| HCC serum (n=61) | Gender | Male | 52 | 85.2 |
|  |  | Female | 9 | 14.8 |
|  |  |  |  |  |
|  | TNM stage | I | 50 | 82.0 |
|  |  | II | 3 | 4.9 |
|  |  | III a | 4 | 6.6 |
|  |  | III b | 1 | 1.6 |
|  |  | ND | 3 | 4.9 |
|  |  |  |  |  |
|  | Grade of differentiation | G1 | 3 | 4.9 |
|  |  | G2 | 12 | 19.7 |
|  |  | G3 | 22 | 36 |
|  |  | ND | 24 | 39.4 |
|  |  |  |  |  |
|  | Age | <40 | 4 | 6.6 |
|  |  | 41-50 | 20 | 32.8 |
|  |  | 51-60 | 26 | 42.6 |
|  |  | >60 | 11 | 18.0 |
|  |  |  |  |  |
| Healthy serum (n=90) | Age | <40 | 5 | 5.6 |
|  |  | 41-50 | 25 | 27.8 |
|  |  | 51-60 | 49 | 54.4 |
|  |  | >60 | 11 | 12.2 |

^a^ND, not determined; G1, high grade; G2, moderate grade; G3, poor grade.
